# Multi-faceted enhancement of full-thickness skin wound healing by treatment with autologous micro skin tissue columns

**DOI:** 10.1038/s41598-021-81179-7

**Published:** 2021-01-18

**Authors:** Christiane Fuchs, Linh Pham, Jermaine Henderson, Katherine J. Stalnaker, R. Rox Anderson, Joshua Tam

**Affiliations:** 1 Wellman Center for Photomedicine, Massachusetts General Hospital, Thier 2, 50 Blossom Street, Boston, MA 02114 USA; 2 Department of Dermatology, Harvard Medical School, Boston, MA USA

**Keywords:** Regenerative medicine, Tissue engineering

## Abstract

Impaired wound healing is an immense medical challenge, and while autologous skin grafting remains the “gold-standard” therapeutic option for repairing wounds that cannot be closed by primary or secondary intention, it is limited by substantial donor site morbidity. We previously developed the alternative approach of harvesting full-thickness skin tissue in the form of “micro skin tissue columns” (MSTCs), without causing scarring or any other long-term morbidity. In this study we investigated how MSTC treatment affects the different cellular processes involved in wound healing. We found that MSTC-derived cells were able to remodel and repopulate the wound volume, and positively impact multiple aspects of the wound healing process, including accelerating re-epithelialization by providing multiple cell sources throughout the wound area, increasing collagen deposition, enhancing dermal remodeling, and attenuating the inflammatory response. These effects combined to enhance both epidermal and dermal wound healing. This MSTC treatment approach was designed for practical clinical use, could convey many benefits of autologous skin grafting, and avoids the major drawback of donor site morbidity.

## Introduction

As the body’s primary physical barrier against the outside world, the skin is regularly subjected to various degrees of injury. In otherwise healthy individuals, skin wounds less than about 1 mm deep are generally able to spontaneously regenerate through a healing process involving four orderly, overlapping phases—hemostasis, inflammation, proliferation, and remodeling^1^. However, wounds that extend into the deeper dermis heal slowly and with both contraction and scarring, leading to permanent impairments in structure, function and appearance^2^. Wounds in skin that is compromised by underlying pathologies, such as vasculopathies or diabetes, often fail to heal, resulting in chronic ulcers. Skin wounds represent an immense challenge to our medical system, annually affecting millions of patients, at tens of billions of dollars in medical expenses, in the US alone^3^. Severe skin wounds can cause critical systemic pathologies, but even wounds that are not immediately life-threatening can have substantial negative impacts on quality of life^4,5^, and certain wound types are associated with 5-year mortality rates that exceed those of many forms of cancer^6–8^. This burden is expected to continue rising in the foreseeable future due to an aging population and the ongoing obesity/diabetes epidemic. Skin scarring is also a substantial medical problem in its own right, as it can lead to long-term medical (e.g. pain, pruritus, reduced range of motion) and psychosocial impairments^9^. There is clearly a need for new therapeutic approaches to improve skin wound healing.

For wounds that cannot be closed by primary or secondary intention, autologous split-thickness skin grafting (STSG) has long been the gold standard, as it is effective at providing a source of epithelium for both acute and chronic wounds^10^. However, skin tissue harvesting for STSG causes substantial morbidity at the donor site, including persistent pain, itching, scarring and disfigurement which patients often find more problematic than the primary wound sites themselves^11^. Intensive research over the past several decades to develop novel “skin substitutes” that could negate the need to harvest donor skin tissue has had limited clinical success. A major reason for this is because skin is a complex tissue, with a host of different interacting cell types, microscale structures such as glands and hair follicles, and extracellular constituents coordinating to perform a wide range of diverse functions. Our understanding of these components and their interactions is still relatively limited, with new discoveries regularly being made that upend conventional understanding. So it is not surprising that efforts to reproduce the natural complexities of skin tissue have been fraught with challenges, and autologous skin grafting remains the only therapeutic option capable of “replacing like with like”.

As an alternative solution, we developed the approach of harvesting full-thickness skin tissue in the form of “micro skin tissue columns” (MSTCs). Even when large numbers (hundreds–thousands) of MSTCs are harvested, the small size (< 1 mm diameter) of each MSTC enables the donor site to heal rapidly, with minimal and transient pain, and without scarring or any other long-term morbidity^12^—findings that have been validated independently in humans^13,14^. This minimization of donor site morbidity effectively eliminates the primary drawback of autologous skin grafting. We also found that diverse cell types and functional skin adnexa from human MSTCs are able to stably engraft in an in vivo model^15^. However, whether/how MSTC treatment affects the different cellular processes involved in wound healing is not known. In the present study, we found that MSTCs are able to enhance multiple components of the wound healing process, including accelerating re-epithelialization by providing multiple cell sources throughout the wound area, increasing collagen deposition, enhancing dermal remodeling, and attenuating inflammatory response. These effects combine to positively alter the trajectory of wound healing.

## Results

### MSTC treatment accelerated re-epithelialization and reduced wound contraction

**Figure 1:**
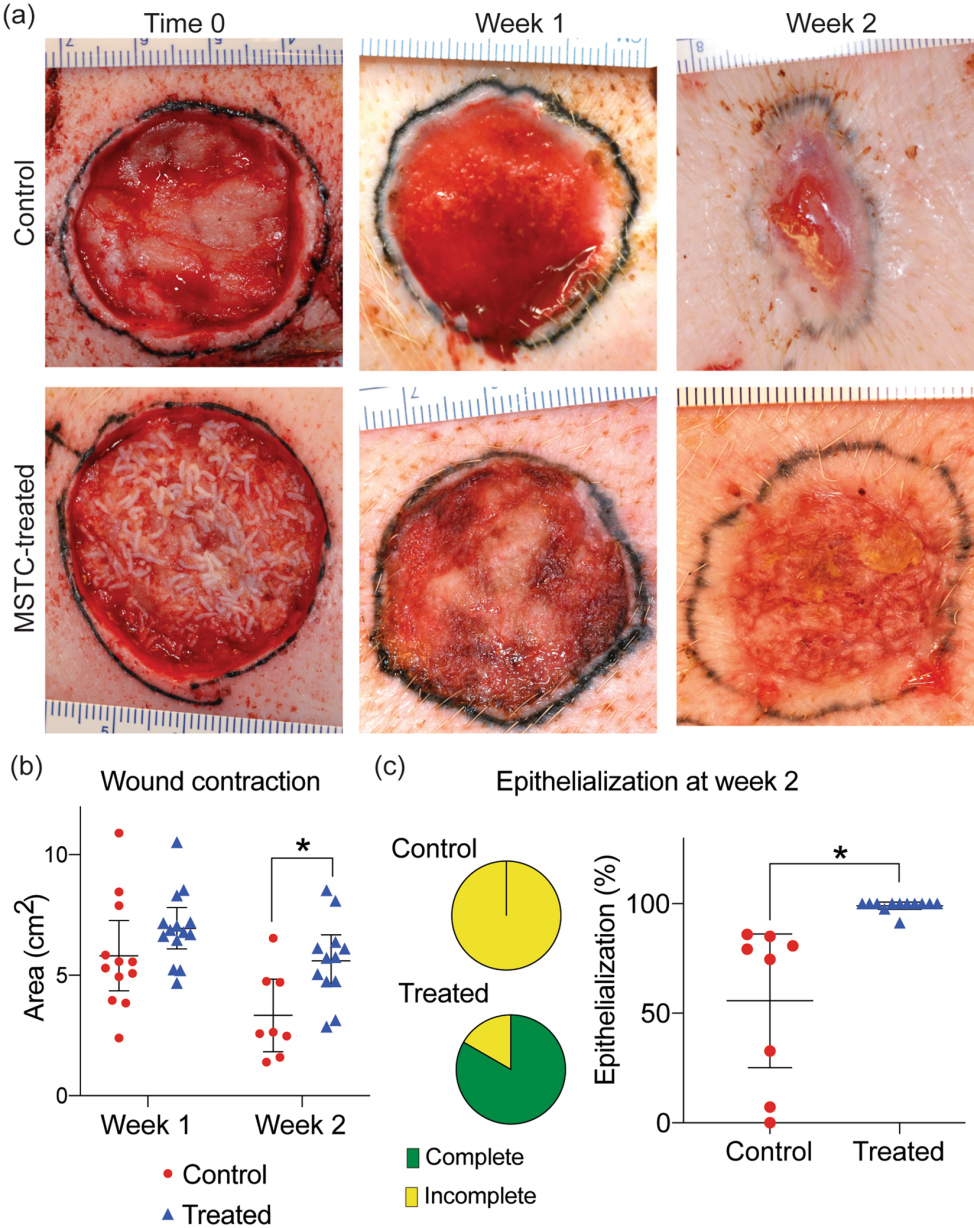
Accelerated re-epithelialization and reduced contraction in wounds treated with MSTCs. (**a**) Representative images of control and MSTC-treated wounds shown at time 0 (immediately after treatment, MSTCs can be seen in the wound bed as short, white, rod-like structures), one week and two weeks after wounding. Wound margins were tattooed with black ink. (**b**) Change in total wound area shows reduced wound contraction in MSTC-treated wounds by week 2. (**c**) Wound areas with visible epithelialization at week 2 were quantified and compared. Pie charts show proportion of wounds that were fully vs. incompletely re-epithelialized at week 2. Graph shows overall extent of re-epithelialization. 10 out of 12 MSTC-treated wounds were completely re-epithelialized, for an overall average of 99% re-epithelialization, whereas none of the 8 control wounds had complete re-epithelialization (average re-epithelialization of 56% in controls). *p < 0.05.

The porcine full-thickness skin wound model was chosen for this study due to the many anatomical and biochemical similarities between human and swine skin^16^, and the high concordance between porcine and human wound data^17^. Since many of the key events in wound healing occur during the first days-weeks after injury, we focused our investigation on the first 2 post-wounding weeks. Wounds treated with MSTCs corresponding to approximately 20% of the excised wound mass were compared to control wounds treated conservatively with hydrogel and absorbent dressings. On the macroscopic level, MSTC treatment caused changes to both the speed and pattern of re-epithelialization, which were readily apparent on visual inspection (Fig. 1). By week 1 there were epithelialized areas visible throughout the MSTC-treated wound surface, that were notably not restricted to the wound edges. In contrast, control wounds healing by secondary intention showed little visible re-epithelialization at this time. By week 2 the MSTC-treated wounds had almost completely re-epithelialized—with re-epithelialization completed in 10 of 12 sites, and over 90% in the remaining 2 sites (Fig. 1a,c), whereas control wounds, with epithelium migration restricted to wound edges, were all only partially re-epithelialized (Fig. 1a,c), and with significantly more contraction than their MSTC-treated counterparts (Fig. 1b).

### Tissue reorganization after MSTC treatment enhanced epidermal coverage and differentiation

**Figure 2:**
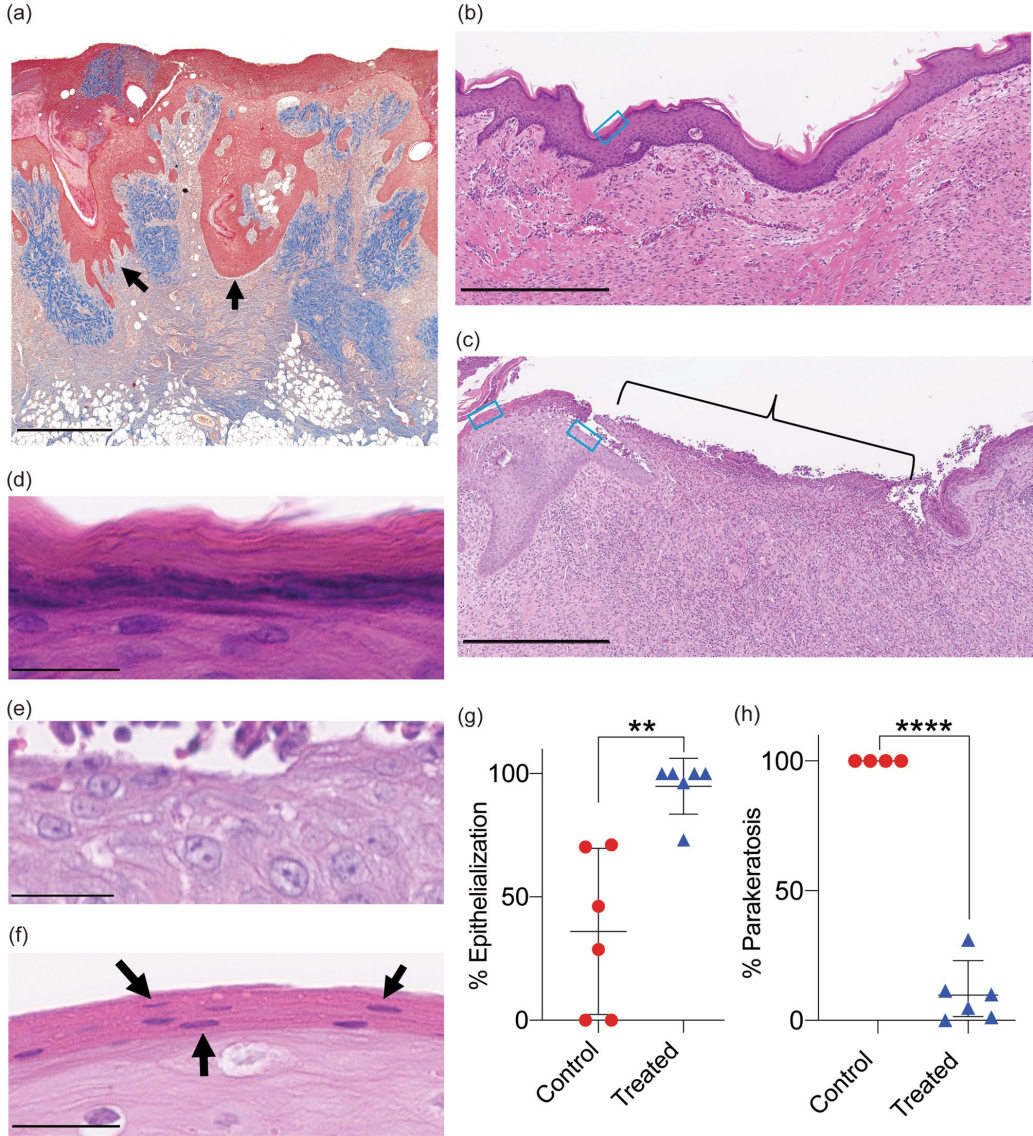
Histologic characterization of wound re-epithelialization. (**a**) Epidermal structures are preserved when using MSTCs to treat wounds. Representative Trichrome image of an MSTC-treated wound after 1 week. Epidermal segments appear to be migrating towards the wound surface, as highlighted by the arrows. Scale bar = 1 mm. (**b**) Representative H&E image of the center of an MSTC-treated wound 2 weeks after treatment, showing complete re-epithelialization. The blue box outlines an example region where the feature highlighted in (**d**) below can be found. Scale bar = 500 µm. (**c**) Representative H&E image of the center of control wounds at 2 weeks. Bracket highlights area without epithelium. The two blue boxes outline example regions where the features highlighted in (**e**) (right box) and (**f**). (left box) below can be found. Scale bar = 500 µm. (**d**) Representative image of the stratum corneum of MSTC-treated wounds, with the characteristic stratified, anuclear structure. Scale bar = 25 µm. (**e**) Representative image of the unepithelialized portion of control wounds. Scale bar = 25 µm. (**f**) H&E image showing parakeratosis (retention of nuclei in the stratum corneum) in epithelialized portions of control wounds. Scale bar = 25 µm. (**g**) Percentage epithelialization and parakeratosis in MSTC-treated (n = 6) and control wounds (n = 6) 2 weeks post wounding are shown in (**g**) and (**h**), respectively. **p $\le$ 0.005, ****p $\le$ 0.0001.

To better understand how MSTC treatment affected wound healing processes at the cellular scale, biopsies were taken from the centers of wound sites at 1 and 2 weeks after treatment and evaluated histologically. At week 1, individual MSTCs were still discernible within the wound beds, but appeared to be in the process of remodeling, with MSTC-derived epidermis in deeper regions of the wound bed migrating towards the skin surface, resulting in “islands” of epithelial coverage forming throughout the wound surface (Fig. 2a). This re-organization of ectopically located epidermal tissue is largely concluded by week 2, resulting in a contiguous epidermis, and complete re-epithelialization of most wounds (Fig. 2b,g). In contrast, since control wounds only re-epithelialized from the wound edges, the centers of control wounds had no epithelial coverage at week 1 (not shown), and gaps in epidermal coverage persisted to week 2 (Fig. 2c,g). In addition, by week 2 the restored epidermis of MSTC-treated wounds was fully stratified, including terminally differentiated stratum corneum through the vast majority of the wound surfaces (Fig. 2d). In control wounds the epidermis was immature, with *stratum corneum* completely lacking in some epidermal regions (Fig. 2e), and parakeratotic in others, with nuclei retained in this normally anuclear skin layer (Fig. 2f,h).

### Increased dermal collagen, reduced myofibroblast density, vascularity, and cellularity

**Figure 3:**
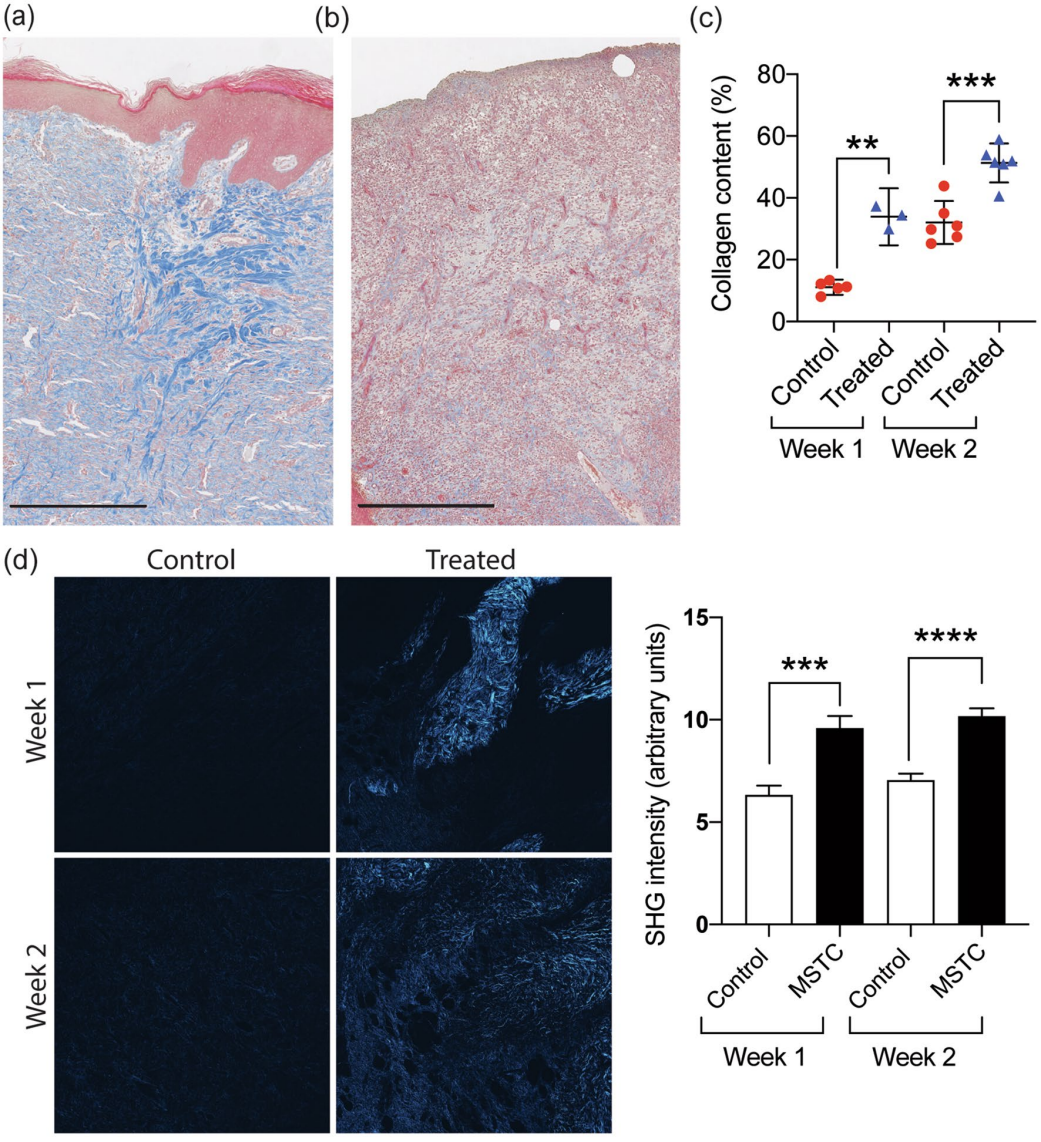
MSTC treatment increased dermal collagen content. Representative trichrome images of MSTC-treated (**a**) and control (**b**) wounds at 2 weeks. Collagen is stained in blue. Scale bars = 500 μm. Quantification of collagen staining shown in (**c**). MSTC-treated wounds had significantly higher collagen content at both 1- and 2-week time points. (**d**) Fibrillar collagen visualized by second-harmonic generation (SHG) imaging. At both time points there were discrete regions with strong SHG signal within MSTC-treated wounds, while SHG signal was largely absent in control wounds. Images shown represent images with intensities that are nearest the mean for each respective group. Each image is 1.9 mm wide. **p $\le$ 0.005, ***p $\le$ 0.0005, **** p $\le$ 0.0001.

**Figure 4:**
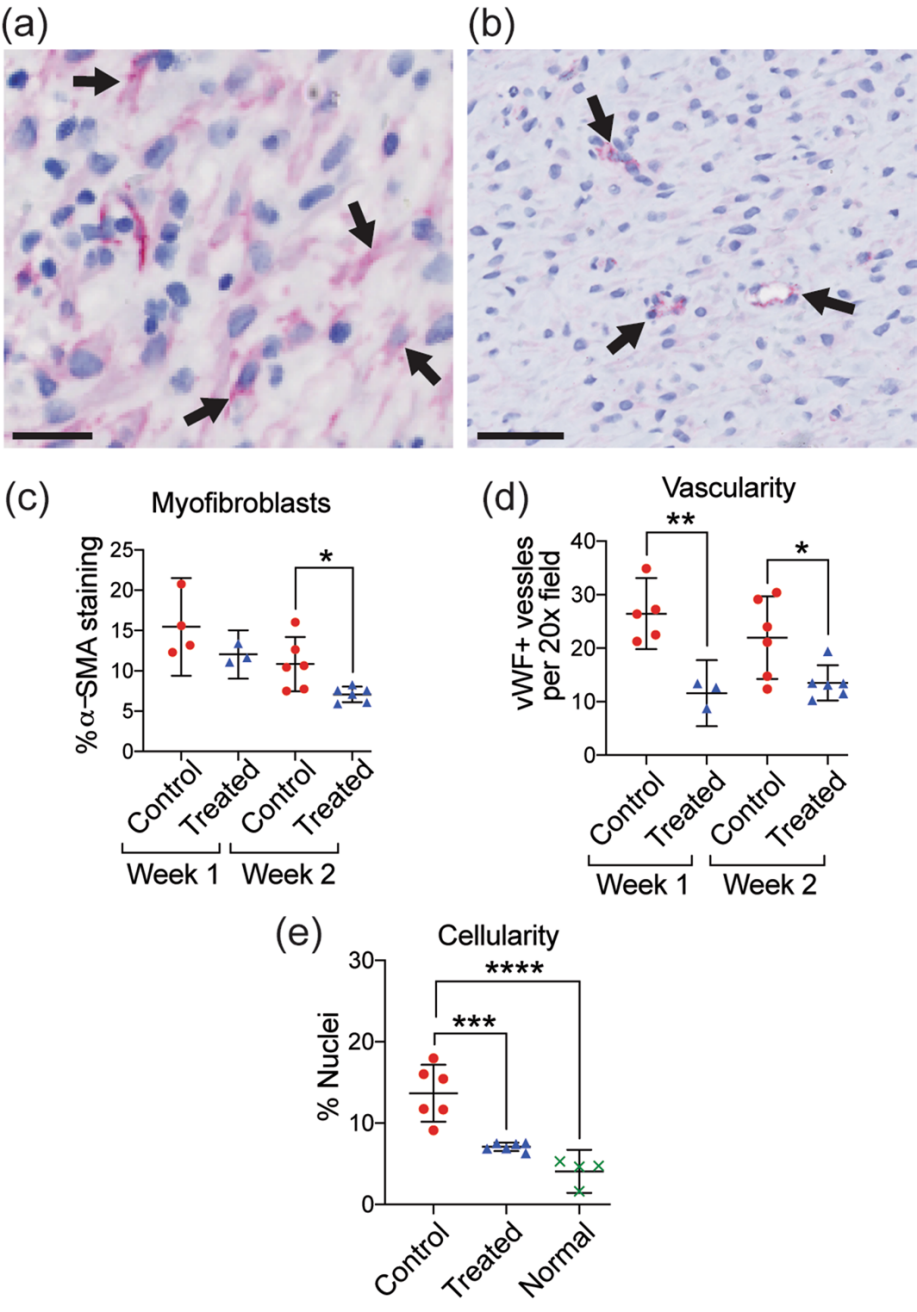
Reduction in myofibroblasts, vascularity, and dermal cellularity in MSTC-treated wounds. Representative images of immunohistochemical staining for α-smooth muscle actin (**a**, myofibroblasts highlighted by arrows, scale bar = 25 μm) and von Willebrand factor (**b**, vascular structures highlighted by arrows, scale bar = 50 μm). MSTC-treated wounds had reduced densities of α-SMA + myofibroblasts after 2 weeks (**c**), as well as vWF + blood vessels at both time points (**d**). Overall cellularity in the dermis was significantly elevated in control wounds at 2 weeks, compared to both MSTC-treated wounds and unwounded normal skin (**e**). *p $\le$ 0.05, **p $\le$ 0.005, ***p $\le$ 0.0005, ****p $\le$ 0.0001.

While re-epithelialization is usually the primary endpoint for wound healing studies, the restoration of dermal structure is arguably a more difficult challenge for deep skin wounds, and the major determinant of the physical quality of the post-healing skin. Since the inclusion of full-thickness dermal tissue is a key feature that distinguishes MSTCs from most other currently available wound treatments, we investigated the dermal remodeling processes after MSTC application. Similar to their epidermal counterparts, the dermal portions of individual MSTCs were still discernable after 1 week, due to their retention of the “basket-weave” collagen pattern that is characteristic of normal dermis, while spaces between MSTCs were filled with granulation tissue (Fig. 2a). By week 2 the MSTC-derived collagen was almost completely integrated within the wound bed, such that individual MSTCs were no longer identifiable. Trichrome staining showed increased collagen content with a more fibrillar morphology in the dermal wound beds of MSTC-treated wounds, compared to controls (Fig. 3a–c). These findings were further validated using second-harmonic generation (SHG) imaging, which showed the presence of fibrillar collagen in the MSTC-treated wounds, but almost none in control wounds (Fig. 3d). The SHG signal in MSTC-treated wounds was mostly clustered in discrete regions, and likely represented collagen present in the original implants (rather than newly produced and organized neocollagen). Control wounds exhibited other hallmarks of deep dermal injury, including formation of highly cellular and vascularized granulation tissue, and the emergence and accumulation of myofibroblasts, which are responsible for wound contraction and rapid (but haphazard) collagen deposition—behaviors that likely conveyed evolutionary advantages, but also increase scarring and dysfunction. In wounds treated with MSTCs the density of myofibroblasts was significantly reduced at 2 weeks in comparison to controls (Fig. 4a,c). In addition, MSTC treatment of wounds also significantly reduced the number of blood vessels at both time points (Fig. 4b,d), and reduced overall cellularity in the dermis at 2 weeks (Fig. 4e).

### MSTC treatment attenuates inflammatory response

**Figure 5:**
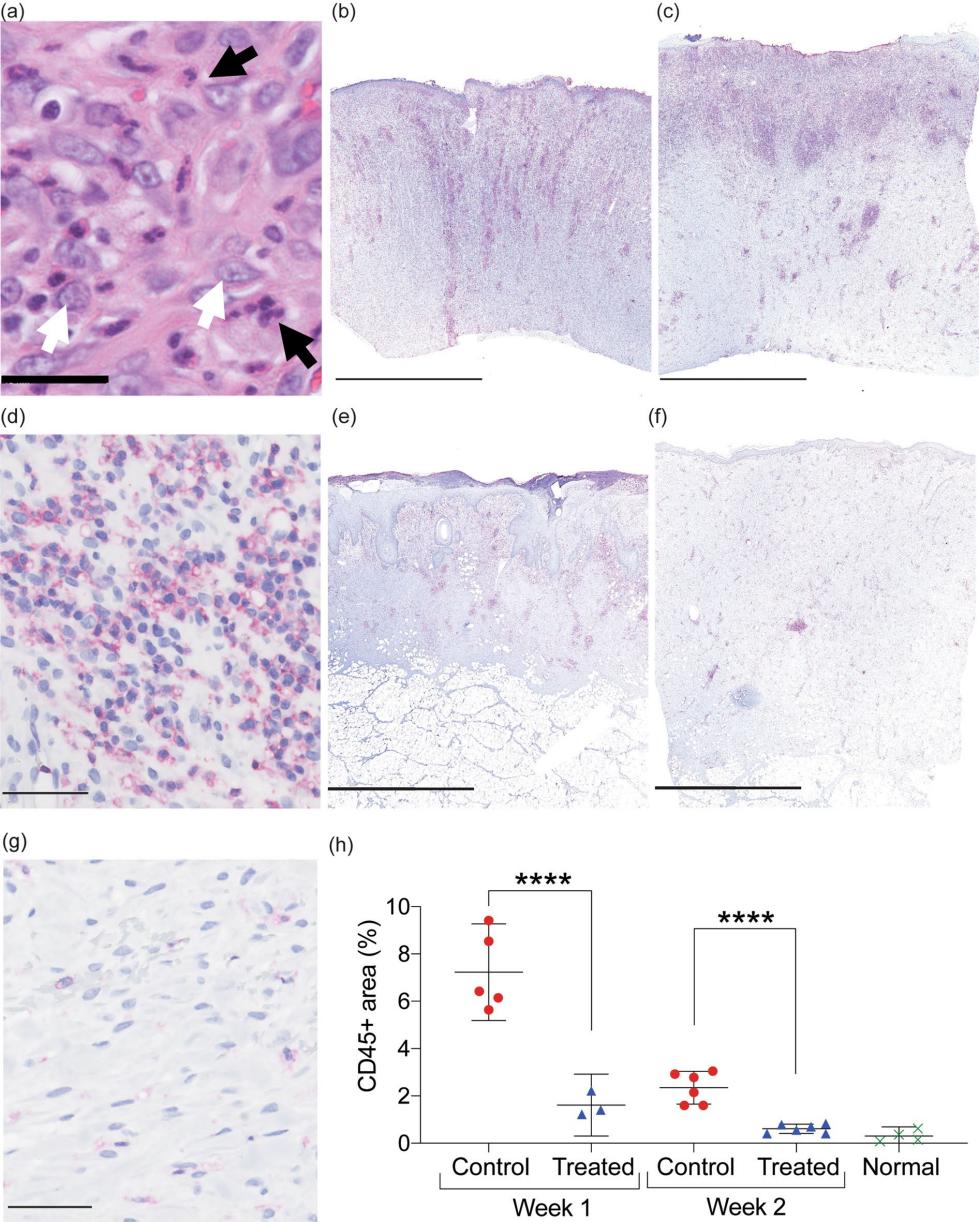
Attenuation of inflammatory response in wounds treated with MSTCs. (**a**) H&E staining showing inflammatory infiltrate in control wounds, with high densities of neutrophils and macrophages (identifiable by their characteristic polymorphic and euchromatic nuclei, respectively). Scale bar = 25 μm. (**b**) Whole-biopsy view of CD45 staining (red) in a control wound at 1 week. Scale bar = 2.5 mm. (**c**) CD45 staining of a control wound at 2 weeks. Scale bar = 2.5 mm. (**d**) High-power view of CD45 staining in a control wound at 2 weeks. Scale bar = 50 μm. (**e**) Whole-biopsy view of CD45 staining of an MSTC-treated wound 1 week. Scale bar = 2.5 mm. (**f**) CD45 staining of an MSTC-treated wound at 2 weeks. Scale bar = 2.5 mm. (**g**) High-power view of CD45 staining in an MSTC-treated wound at 2 weeks. Scale bar = 50 μm. (**h**) Quantitative evaluation of CD45+ areas in MSTC-treated vs. control wounds. Density of CD45+ leukocytes was significantly lower in MSTC-treated wounds compared to controls at both time points. ****p $\le$ 0.0001.

The inflammatory phase of wound healing plays a central role in orchestrating the healing process, and a dysregulated inflammatory phase, such as one that is excessive or fails to resolve in a timely manner, is believed to be responsible for many of the most undesirable healing outcomes^18,19^. Thus, we sought to investigate the effect of MSTCs on the inflammatory response. Control wounds healing by secondary intention were characterized by exuberant inflammatory infiltrates, densely populated by neutrophils and macrophages, at both 1- and 2-week time points (Fig. 5a). Immunohistochemical staining against the pan-leukocyte marker CD45 was used to quantify the extent of inflammatory cell infiltration into the wound sites. Control wounds were extensively infiltrated by CD45+ leukocytes during the first 2 weeks, especially towards the skin surface (Fig. 5b–d). Leukocyte density in MSTC-treated wounds was substantially lower than controls at both time points (by 4.5- and 3.8-folds respectively, Fig. 5e–g), and by week 2 it had returned to levels close to those of uninjured normal skin (Fig. 5h).

### Higher abundance of viable adipocytes in MSTC treated wounds

**Figure 6:**
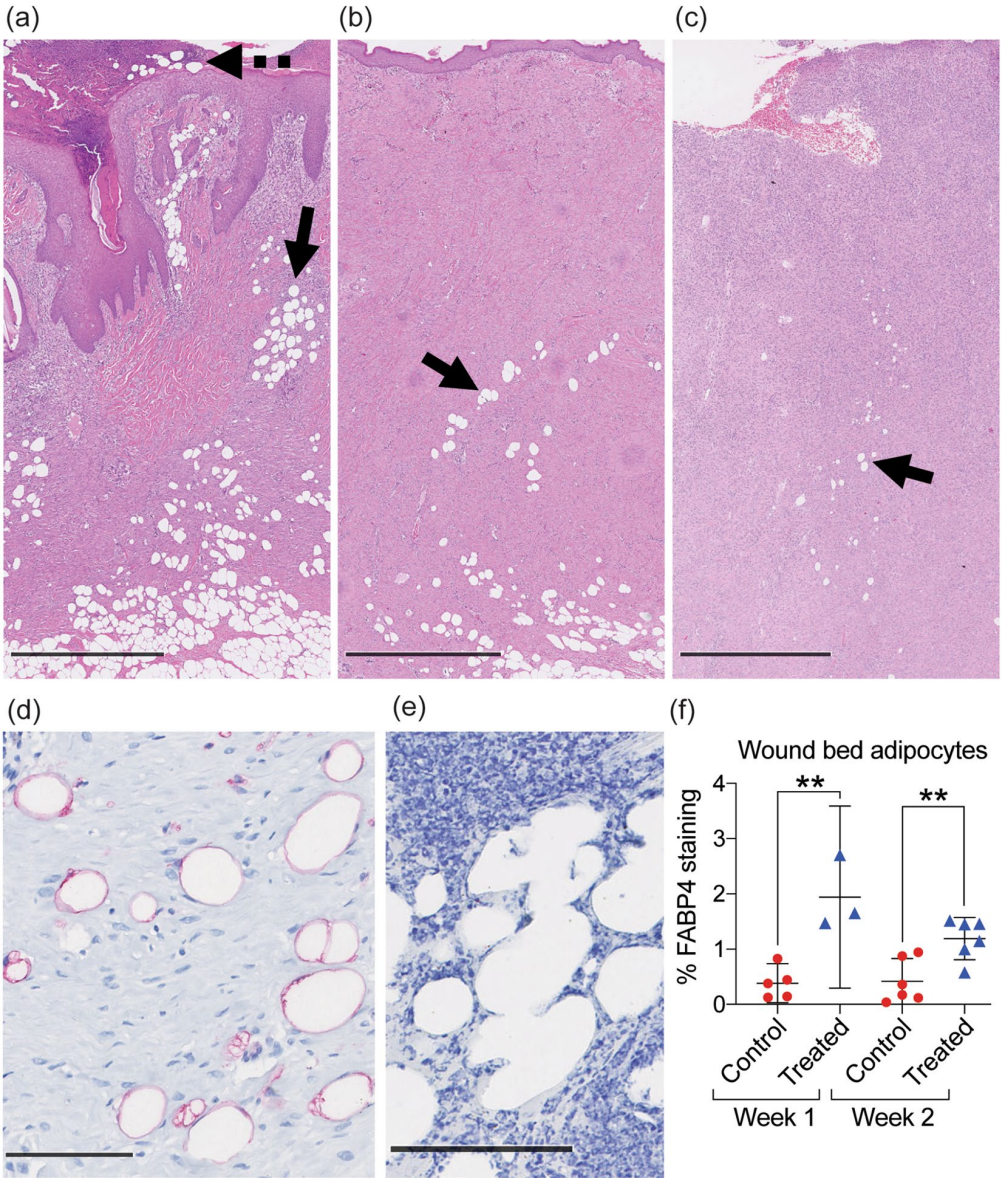
Presence of mature adipocytes in wound beds. (**a**) H&E staining of MSTC-treated wound at 1 week. Many adipocyte-like structures were seen within the wound bed (solid arrow) and outside the epidermal surface (dotted arrow). (**b**) MSTC-treated wound at 2 weeks, showing adipocyte-like structures still present in wound bed (arrow). (**c**) Fewer adipocyte-like structures were also present in some control wounds (arrow). (**a**–**c**) Scale bars = 1 mm. (**d**) IHC staining for the adipocyte marker FABP4 (red). (**e**) Adipocyte-like structures outside of the epidermal surface (dotted arrow in **a**) did not retain FABP4 expression. (**d**,**e**) Scale bar = 100 μm. (**f**) MSTC-treated wounds had higher density of FABP4+ adipocytes than control wounds at both time points. **p $\le$ 0.005.

On detailed examination of histologic samples, we observed structures that resembled adipocytes in most of the wound beds, in both treatment and control groups (Fig. 6), but these were especially prevalent in MSTC-treated wounds (Fig. 6a,b). At the 1-week time point some of these adipocyte-like structures could also be found outside the epidermis (Fig. 6a). Unlike normal intradermal and subcutaneous adipocytes, which generally aggregate in large clusters called lobules, wound bed adipocytes generally appeared as separated individual cells. Immunohistochemical staining against the adipocyte marker FABP4 (Fatty Acid Binding Protein 4) confirmed that most of these structures were indeed differentiated adipocytes (Fig. 6d). Some adipocyte-like structures did not express FABP4, including all of those outside the epidermis (Fig. 6e), these likely represent remnants from nonviable adipocytes. Quantification of FABP4+ adipocytes by digital image analysis confirmed that MSTC-treated wounds were more densely populated by adipocytes at both 1- and 2-week time points (Fig. 6f).

## Discussion

This study shows that MSTC treatment positively impacts multiple components of the wound healing process, including re-epithelialization, dermal matrix deposition, and resolution of wound inflammation, culminating in improvements in both the speed and quality of wound healing.

In this study we compared MSTCs to a hydrogel wound dressing, which is commonly used for wound care, but more complex wound types may require more advanced therapies, of which there is a wide variety currently available, including such disparate options as skin grafting, negative pressure wound therapy, hyperbaric oxygen, shockwave, electrical stimulation, cultured tissue products, and any number of synthetic or human/animal-derived biomaterials. Amongst all these treatment options, only autologous cell/tissue-based treatments are capable of directly supplying new cells to replenish a wound volume, whilst all other treatments work indirectly by attempting to make the wound environment more conducive towards repopulation by host cells migrating in from surrounding, unwounded tissue. Autologous minced skin grafting is the closest analog to our MSTC treatment approach—the main differences being the inclusion of deep dermal tissue and minimization of donor site morbidity in MSTCs. Minced skin grafting has quite a long history, having first been described in the 1950’s^20^, and with its close cousin the pinch graft going back even further to 1869^21^. The increased amounts of graft edges from which cell outgrowth could occur was thought to be advantageous compared to an intact graft of similar total area^20^. Minced skin grafts have been shown to effectively enhance healing in a wide variety of wound types including large burns, traumatic wounds and chronic ulcers^22–24^, and it seems reasonable to expect MSTCs to perform at least comparably in similar settings. The inclusion of deep dermal elements in MSTCs has the potential to confer additional benefits that are not available to minced grafts derived from split-thickness skin, but that remains to be proven in humans (the addition of dermal collagen, as shown in Fig. 3, may provide direct benefits, since collagen-based materials are often used as topical wound treatments^25^, and we previously found that MSTC-derived adnexal structures are able to retain long-term viability and functionality in a rodent xenograft model^15^). Another more recently developed method for applying autologous skin tissue is to enzymatically dissociate it into a single-cell slurry, which is then sprayed over wound beds. While this method could potentially cover large areas with relatively little donor tissue, in a porcine full-thickness excision wound model (similar to the one used in our study), cell spraying alone was unable to restore epidermal coverage within a 3-week period (compared to complete re-epithelialization within 2 weeks with MSTCs), and re-epithelialization was only achieved when combined with the INTEGRA dermal regeneration template^26^. Consistent with clinical experience where a second stage surgery to provide epidermal coverage by split-thickness skin grafting is often necessary after wound bed granulation is induced with INTEGRA, INTEGRA alone was also unable to accelerate re-epithelialization^26^. The same could be expected of most acellular materials, since without additional sources of cell growth within the wound bed, re-epithelialization can only occur from the wound edges. In our study MSTC treatment alone was sufficient to enhance re-epithelialization of full-thickness wounds, which is a significant advantage. The improved outcome when sprayed cells were combined with INTEGRA also points to the potential that similar improvements may be possible by combining MSTCs with other compatible wound therapies.

MSTCs are also expected to share similar limitations as minced skin grafts, in particular since the grafted tissue is not immediately protected by the skin’s barrier function (as it would be for a conventional skin graft), the graft tissue is susceptible to desiccation, and must be protected by additional dressing regimens to keep the wound sites hydrated until barrier function is re-established at the graft site^27,28^ (within 2 weeks in our model). In addition, wound beds that are hostile to tissue grafting, e.g. due to ischemia or high microbial burden, would likely also present challenges for treatment with MSTCs.

Since the MSTCs are applied to wound sites “randomly”, i.e. without maintaining the epidermal-dermal skin polarity, cells/structures associated with the MSTCs are likely to be ectopically located upon application into wound sites, and must migrate and reorganize to restore the overall skin architecture. We found that epidermal keratinocytes were able to complete this reorganization within 2 weeks to form a contiguous, fully stratified epidermal layer. This is consistent with previous reports of keratinocytes being able to migrate to and re-epithelialize wound surfaces following implantation of split-thickness skin grafts either upside-down or in a minced, randomly-oriented form, as long as a moist wound environment was maintained^27,28^. The ability of MSTCs to serve as cell sources for re-epithelialization shifts the healing process of full-thickness wounds (which can normally only re-epithelialize from the wound edges) to behave more like partial-thickness wounds (where re-epithelialization can occur throughout the wound area, with surviving adnexal structures in the deep dermis serving as epithelial cell sources^2^). This particular advantage of MSTC treatment is likely to be more prominent with larger wound areas, as the area-to-perimeter ratio increases.

The reorganization of dermal skin components is a more complicated matter. Whereas the epidermis has many characteristics that are naturally conducive to tissue repair/regeneration, including a relatively simple, homogenous, and avascular architecture, little extracellular matrix, and a high baseline cell turn-over rate, the dermis is highly heterogenous in both structure and composition, harboring complex multicellular structures such as pilosebaceous units, sweat glands, vasculature, and neuronal networks, as well as an intricately structured extracellular matrix that gives rise to much of the skin’s mechanical properties. Many of these dermal components are naturally long-lasting and difficult to regenerate in human adults, and their absence or disruption underlies much of the pathologic characteristics of skin scars. Skin fibroblasts were once considered a homogenous and rather nondescript cell type, but it is now clear that there are multiple distinct lineages of skin fibroblasts, which differ based on skin depth and anatomic location, and that play different roles in tissue homeostasis, wound healing, and scarring^29–31^. Similarly, wound-associated myofibroblasts also consist of different subtypes, including populations that are transdifferentiated from other origins, such as myeloid cells^31^ and adipocyte precursors^32^. In many cases these different populations and their respective functions are still being identified, so it is perhaps not surprising that even after decades of concerted effort in the field, it is still not possible to recreate the dermis artificially. In this study we did not attempt to identify or track the fate of these different subpopulations of dermal cells, since validated molecular markers are mostly not yet available for the swine. However, since each MSTC consists of a random sampling of full-thickness skin tissue, MSTCs in aggregate should contain all the cellular and extracellular components of natural dermis. This is a key feature distinguishing MSTCs from split-thickness skin grafts (STSGs), STSG-based wound applications (e.g. STSGs applied in minced or dissociated single-cell form), or cultured tissue products. The changes in dermal characteristics caused by MSTC treatment, including increased collagen content, reduced myofibroblast density, vascularity, and cellularity, are all consistent with MSTCs contributing to dermal wound remodeling and progression towards more normal dermal features, compared to control wounds which were still largely occupied by granulation tissue at the study timepoints.

In this study we applied MSTCs at approximately 20% of the excised mass for each wound. Assuming similar skin thickness between harvest and recipient sites, the mass of the implanted tissue would scale with its epidermal surface area, such that a 20% replacement for a 3 cm-diameter recipient wounds would correspond to approximately 370 individual MSTCs. Harvesting hundreds-thousands of individual implants manually (as we did in this study) would be impractical in clinical settings, but a commercial device that is under development to apply this technology in humans is capable of harvesting over 300 implants within a few seconds, and an early version of this device has already been used in pilot clinical trials^13,14^. We have not addressed the issue of “dosage” in this study. Previous work by others have shown that minced skin grafts can be applied at up to 100-fold expansion ratios and still achieve clinical benefit^33^, so it seems likely that MSTCs could be applied at a lower density if necessary. Conceptually, while larger amounts of “starting material” provided by higher grafting densities may have obvious benefits, at some point these may be counteracted by the increased demands on oxygen and nutrients, and potentially difficulties in cellular reorganization in an increasingly crowded wound bed. The optimal amount of graft tissue is likely to be dependent both on donor tissue availability, and on wound parameters such as dimensions, extent of granulation, and vascularity.

The ability of MSTCs to deliver normal dermal tissue into the wound bed is likely to be a critical factor in this capacity to enhance dermal remodeling, via a number of potential mechanisms. The increased number and heterogeneity of dermal fibroblast subtypes conveyed by MSTCs are likely to affect both the amount and architecture of collagen deposition. The presence of normal dermal cells and extracellular matrix may lead to a different paracrine signaling milieu in the wound bed that could impact multiple dermal remodeling events, such as emergence/behavior of myofibroblasts, and construction of new dermal extracellular matrix. The extracellular matrix components from MSTCs may also directly contribute to the construction of the neodermis, as it has been previously reported that about 90% of the dermal portions of minced STSGs were incorporated into the newly formed dermis (with the remaining 10% expelled through the neoepidermis)^34^. The reduced wound contraction is another indication that the dermal components in MSTC are able to impact wound remodeling, since wound contraction is inversely proportional to the amount of dermal tissue included in skin grafts^35^. The ability to reduce wound contraction is also a significant clinical advantage, since contracture can lead to significant functional impairments that often require additional surgical interventions.

In this study we have focused on the early effects of MSTC treatment—primarily during the inflammatory and proliferative phases of the classical 4-staged wound healing process (hemostasis, inflammation, proliferation, and remodeling). We have previous shown that the reduced wound contraction persists for at least 8 weeks in the porcine model^12^, but whether/how the various MSTC-related effects reported in this study would lead to long-term improvements in the structure and function of MSTC-treated skin wounds, would have to be evaluated in future clinical studies, since the remodeling phase of wound healing can continue for years.

While the presence of adipocytes is expected immediately after the wounds were treated with “randomly” oriented MSTCs, it was surprising to find viable adipocytes (evidenced by continual FABP4 expression) persisting in the wound beds after 2 weeks, when the initial expulsion of dermal fragments had already taken place—a process that was estimated to eliminate about 10% of randomly-implanted dermal tissue in a previous study^34^. Adipose-derived stem cells have long been recognized for their ability to augment tissue healing/regeneration, and in recent years there has been accumulating evidence that fully-differentiated, mature adipocytes can also impact wound repair in many ways, including regulating hair follicle regeneration, modulating keratinocyte and fibroblast behavior, initiate and regulate inflammatory processes, and producing antimicrobial peptides^36,37^. In Drosophila, adipocytes have even been reported to actively migrate into wounds and participate in clearance of wound debris and sealing of the epithelial wound gap^38^. The presence of FABP4+ adipocytes in the wound beds of even control wounds closing by secondary intention suggest that these cells may be capable of similar behaviors in large animals. MSTCs contain both intradermal and subcutaneous fat, which we showed are transferred into the treated wounds where at least some are able to persist through the re-epithelialization process. The function of these fat cells, and whether they originate from MSTCs from the initial transplantation, differentiate from adipocyte progenitors, or are recruited from the existing subcutaneous fat by MSTC-derived secreted factors, remains to be elucidated.

The inflammatory response is a pivotal component of the wound healing process^19^. Under normal circumstances, skin injuries are closely followed by a spike in inflammation to defend against infectious organisms and remove wound debris, then inflammation subsides and transitions from a proinflammatory to a reparative status, which is critical for enabling the subsequent tissue repair processes. Finally, inflammatory cells are eliminated from the wound site during the later stages of healing via reverse migration or apoptosis. Dysregulation of this inflammatory response is a major contributor to both fibrotic scarring and chronic ulceration. We found that MSTC treatment significantly hastened the resolution of wound inflammation. Relatively little is known about the cellular and molecular mechanisms governing the resolution of wound inflammation, but the spatial distribution of leukocytes, which were most densely populated near the skin surface, suggests that epidermal restoration may be a significant factor in modulating the inflammatory response. This may be largely due to reduced exposure to external environmental agents and pathogens, but signaling from MSTC-derived cells may also be a significant factor, as keratinocytes, dermal fibroblasts, and adipocytes are all known to actively influence inflammatory events^32,39–41^. This MSTC-mediated attenuation of the inflammatory response may have long-term implications on the quality of the post-healing tissue, as excessive inflammation is believed to be a significant contributor to skin scarring^19^. Chronic wounds are also characterized by dysregulated, persistent inflammation, which our data suggests may be ameliorated by MSTC treatment. Since current animal models are unable to faithfully replicate either scarring or chronic wounds as they occur in humans, these potential effects of MSTC treatment will also be best studied in clinical trials.

In summary, this study shows that MSTCs improve multiple aspects of the wound healing process, enhancing both epidermal and dermal wound healing. This treatment approach was intentionally designed to be practical for patients, with fast technically straightforward harvesting under local anesthesia, simple transfer and application of MSTC to a wound, and far less morbidity in the donor site compared conventional skin grafts. Harvesting and transfer of MSTC also avoids in vitro cell cultures and the use of exogenous materials—this is an entirely “bedside” procedure that transfers many small pieces of full-thickness skin into a wound. These characteristics should make MSTC grafting a valuable addition to the armamentarium for wound care.

## Materials and methods

### Animal procedures

All animal procedures were performed in accordance with the Public Health Service Policy on Humane Care and Use of Laboratory Animals, and with approval by the Massachusetts General Hospital Institutional Animal Care and Use Committee. Adult female Yorkshire swine, ~ 35 kg at time 0, were used. Custom-made harvesting needles (19 G) were used to harvest full-thickness micro skin tissue columns (MSTCs) from the animal’s rump as described previously^12,42^. After MSTC collection, a series of 3 cm diameter, full-thickness skin wounds were produced on the trunk skin of the animals by excision down to the subcutaneous fat. Wound margins were marked with tattoos. MSTCs corresponding to approximately 20% of the excised skin mass were randomly scattered over the wound bed. Wounds that were allowed to heal by secondary intention served as controls. Treatment and control wounds were placed in an alternating spatial pattern to control for anatomical variations in skin properties. All wounds were dressed by a combination of hydrogel and foam dressings (Tegaderm, 3 M Health Care, St. Paul, MN)—a regimen previously shown to enable keratinocyte reorganization after randomized application of minced STSG fragments^27^. The dressed wounds were additionally protected by an adherent dressing (DuoDERM CGF, ConvaTec, Greensboro, NC), followed by stockinette and a nylon jacket. A total of 12 MSTC-treated wounds and 11 controls, from 4 different animals were used. Wounds were inspected and photographed after 1 and 2 weeks. 10-mm biopsies were collected from wound centers at each time point for histological analysis. Each wound was only biopsied once.

### Gross image quantification

To quantify wound contraction, a ruler was included in each wound photograph, then the wound outline was traced digitally and the ruler in each image was used to convert pixel values to physical areas using Fiji software. Similarly, the extent of re-epithelialization was assessed by tracing then measuring wound areas that appeared open (based on the distinctive glistening appearance of areas lacking epithelium) in each image.

### Histology

Skin biopsies were fixed in 4% formaldehyde, embedded in paraffin, cut into 5 μm sections, then stained with hematoxylin and eosin (H&E) for general histology and Gömöri's Trichrome for collagen. Immunohistochemical staining following a previously described protocol^15^, with primary antibodies against α-smooth muscle actin (α-SMA, for myofibroblasts, 1:100, ab5694, Abcam, Cambridge, MA), CD45 (for leukocytes, 1:2000, ab10558, Abcam), von Willeband factor (vWF, for endothelial cells, 1:2000, A008229-5, Agilent, Santa Clara, CA) and Fatty Acid Binding Protein 4 (FABP4, for adipocytes, 1:1000, LS-C293834, LSBio, Seattle, WA), was used to detect expression of the respective markers. Stained slides were scanned into digital format using the Nanozoomer (Hamamatsu, Bridgewater, NJ). Representative whole-biopsy images for each experimental group and time point are included in the Supplementary Information.

#### Second-harmonic generation imaging

Second-Harmonic Generation (SHG) imaging was performed on paraffin tissue sections (5 μm, label-free), using an inverted multiphoton laser scanning confocal microscope, FluoView FV-1000 MPE (Olympus, Central Valley, PA, USA), equipped with a MaiTai HP DeepSee titanium:sapphire mode-locked two-photon laser, tunable 690–1040 nm (Newport/Spectra-Physics, Santa Clara, CA, USA). The Ti:Sapphire femtosecond laser was tuned to 900 nm for two-photon excitation and the emission was passed through a red dichroic mirror (RDM 690 nm). The two-photon laser illumination is circularly polarized and the detection is backward propagation (reflected). The backward scatter SHG signals and the autofluorescence (AF) signals were collected through a 425/30-nm and a 525/45-nm emission band pass filter, respectively, with a dichroic mirror 485 nm to separate them. An Olympus UPLANSAPO 20 × objective (N.A. = 0.75, WD 0.6 mm), was used to focus the excitation beam and to collect the SHG and AF signals.

Regions of interest (ROIs) were randomly selected within the dermal wound regions from each tissue section, by operators who were blinded to the treatment conditions. For each ROI, multiple tiled images were captured with a motorized stage, and were stitched together after image acquisition square images of 1903 × 1903 μm, at 6139 × 6139 pixels of resolution. Image acquisition and processing were performed with the Olympus Fluoview FV 10 ASW 4.1 software^43^. SHG signal intensity was quantified using Fiji^44^.

### Image quantification

For the quantification of histological features and IHC staining, at least 4 non-consecutive sections, each 100 μm or more apart, were taken from each tissue sample. Digital image quantification was performed using NDP.view software (Hamamatsu) and Fiji^44,45^, as detailed in the Supplementary Information.

### Statistical analysis

For statistical analysis, each individual wound site was considered an independent sample, with n = 3–5 at week 1, and n = 6 at week 2. Results from quantification of histologic samples were averaged for each wound site, the means were then used for analysis using Graph Pad Prism version 8.3.1 for macOS (GraphPad Software, San Diego, California). Comparisons between control and treated sites were made using the two-tailed Student’s t-test. Comparisons between multiple groups were made using ordinary one-way ANOVA with Tukey’s post-hoc test. *P*-values ≤ 0.05 were considered statistically significant. Individual data points as well as mean ± 95% confidence intervals are shown in the figures.

## Supplementary Information

Supplementary Information.
