# Supplementary material for: Multi-faceted enhancement of full-thickness skin wound healing by treatment with autologous micro skin tissue columns
Source: Sci Rep. 2021 Jan 18;11:1688. doi: 10.1038/s41598-021-81179-7 (PMC7814113; doi:10.1038/s41598-021-81179-7)
Supplement: Supplementary file 1 — Supplementary Information. [file 41598_2021_81179_MOESM1_ESM.docx]

Title: Multi-faceted enhancement of full-thickness skin wound healing by treatment with autologous micro skin tissue columns

Authors:

Christiane Fuchs^1,2^, Linh Pham^1^, Jermaine Henderson^1^, Katherine J. Stalnaker^1^, R. Rox Anderson^1,2^, Joshua Tam^1,2^*

Affiliations:

^1^Wellman Center for Photomedicine, Massachusetts General Hospital, Boston, MA.

^2^Department of Dermatology, Harvard Medical School, Boston, MA.

* To whom correspondence should be addressed: Wellman Center for Photomedicine, Thier 2, Massachusetts General Hospital, 50 Blossom Street, Boston MA 02114.

Telephone: 617-724-3955

Email: [jtam3@mgh.harvard.edu](mailto:jtam3@mgh.harvard.edu).

**Supplementary Materials**

**Image quantification**

Quantification of the degree of epithelialization on the histologic level was done by using NDP.view software (Hamamatsu) to trace both the epithelialized and total wound surface of each H&E-stained biopsy sample, then the % epithelialization was calculated from the ratio between the two. Similarly, the % parakeratosis was calculated from the ratio between the length of stratum corneum that retained nuclei, versus total stratum corneum surface length (wound areas without stratum corneum coverage were not considered in calculating % parakeratosis). All other image processing operations were performed with Fiji (Schindelin et al., 2012). For the quantification of trichrome, nuclei, CD45, α-SMA, and vWF staining, 4 – 8 regions of interest (ROIs) were randomly and blindly selected at 20x magnification from each tissue section. Since individual MSTCs were still discernable at week 1, for MSTC-treated wounds at 1 week ROIs were selected outside of areas occupied by MSTCs, so that the analysis only included the wound beds, not the implanted tissue. For week 2 samples, ROIs were selected randomly throughout each biopsy (since the biopsies were taken from the center of each wound, there was no risk of confounding effects from normal unwounded tissue). The color deconvolution tool in Fiji (Ruifrok and Johnston, 2001) was used to extract the target staining colors, the images were then thresholded to identify areas with positive staining (Supplementary Fig. 1). For trichrome, CD45, and α-SMA staining, the % positive staining was calculated as the ratio between area with corresponding staining versus total ROI area. The number of vWF+ vessels in each field was counted manually.


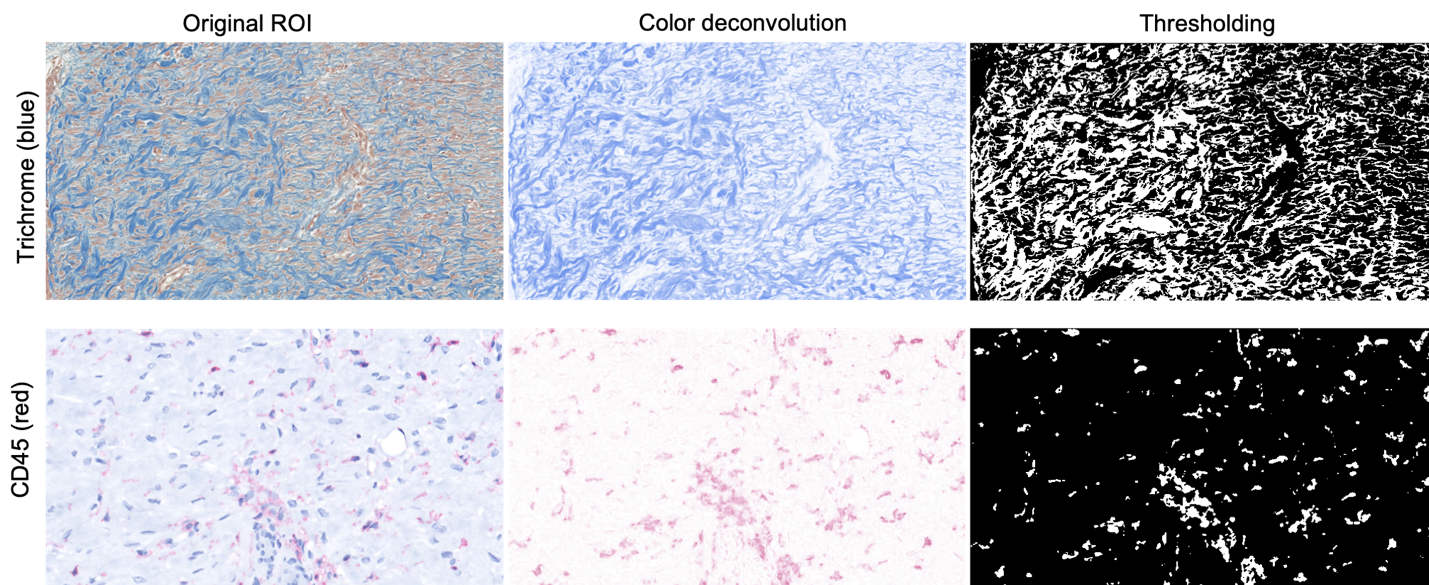


***Supplementary Figure 1:* Quantitative image analysis.** Two examples (trichrome – top row, and CD45 – bottom row) are shown to illustrate the quantitative image analysis process. Color deconvolution is used to extract the staining color of interest. The image is then binarized by thresholding. Finally, the area occupied by the target stain (white areas in the images in the “Thresholding” column) is measured and compared to the area of the whole ROI to obtain percentage staining.

The quantification of FABP4 staining was performed differently, since the staining pattern was relatively sparse and heterogenous. Instead of ROIs, images of the entire wound area were analyzed. First the areas containing normal subcutaneous fat (identifiable by anatomic location as well as the characteristic tight clustering of adipocytes) were excluded. Then areas with open negative space were obtained by image thresholding. Areas with FABP4+ staining were again obtained by color deconvolution followed by thresholding. The two sets of images were contrasted to identify areas where FABP4 staining overlapped with open negative space, corresponding to mature unilocular adipocytes (Supplementary Fig. 2). The % adipocyte was calculated as the ratio of this area versus the total wound area.


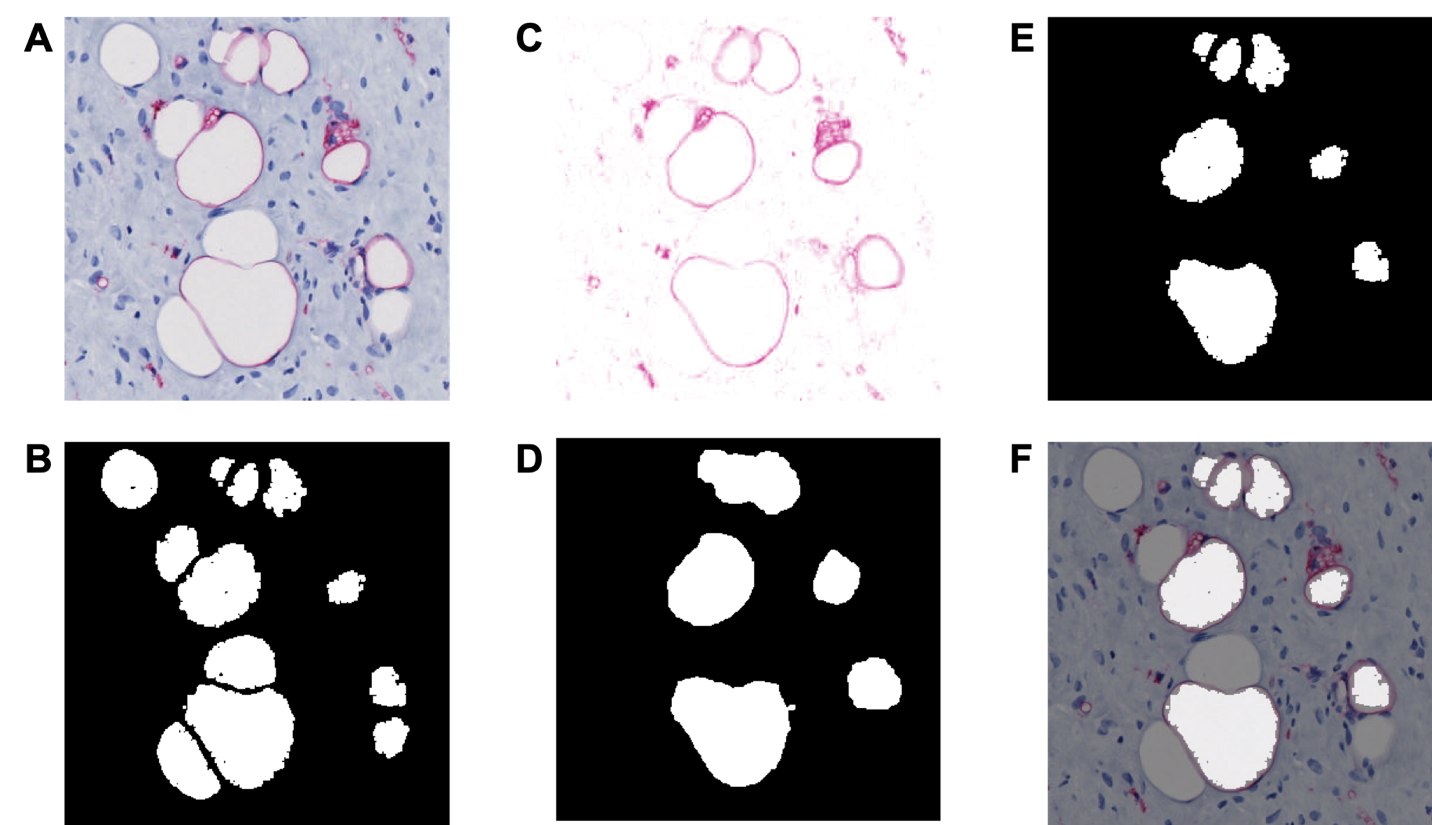


***Supplementary Figure 2:* Quantitative image analysis for adipocyte staining.** A different algorithm was used to quantify the amount of adipocytes in the wound beds, due to the unique morphology of mature adipocytes: a thin cytoplasm surrounding a unilocular lipid store that appears as negative space on histology, such that the positive staining area alone does not reflect the area occupied by an adipocyte. In this algorithm, the original immunohistochemistry image (A) is first thresholded to obtain the negative space (B). The staining color (red) is then extracted by color deconvolution (C), then the image is processed through a series of thresholding, dilation, skeletonization, hole filling, and opening operations, to obtain areas that are surrounded by FABP4 staining (D). The binary images for negative space (B) and areas surrounded by FABP4 staining (D) are then compared to return an image of areas with both open negative space and surrounding by FABP4 staining (E), which corresponds to mature adipocytes (F, showing image E overlaid on image A).

***Supplementary Figure 3:* Whole-biopsy image of control wound at week 1:**


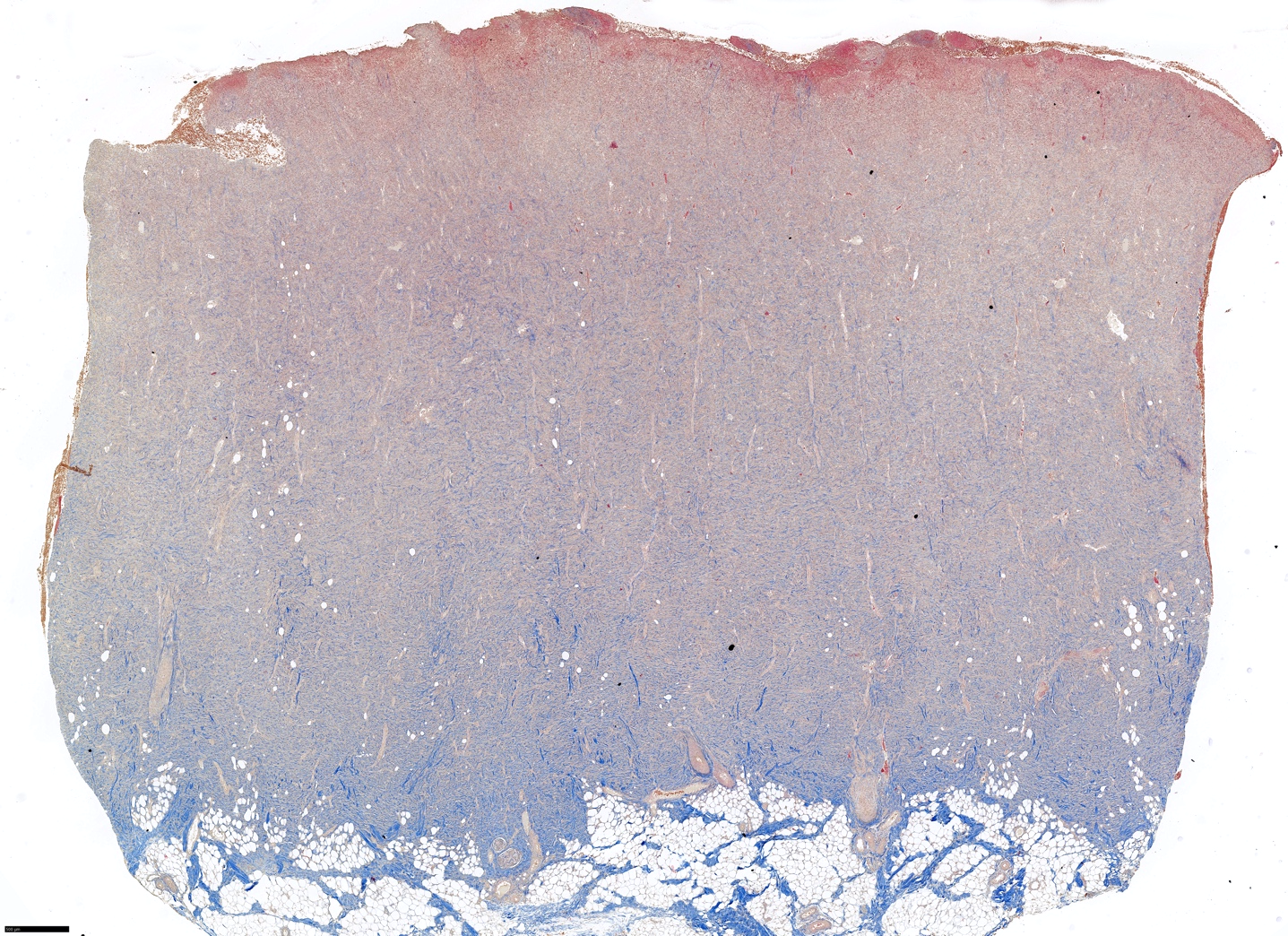


500 μm

***Supplementary Figure 4:* Whole-biopsy image of MSTC-treated wound at week 1:**


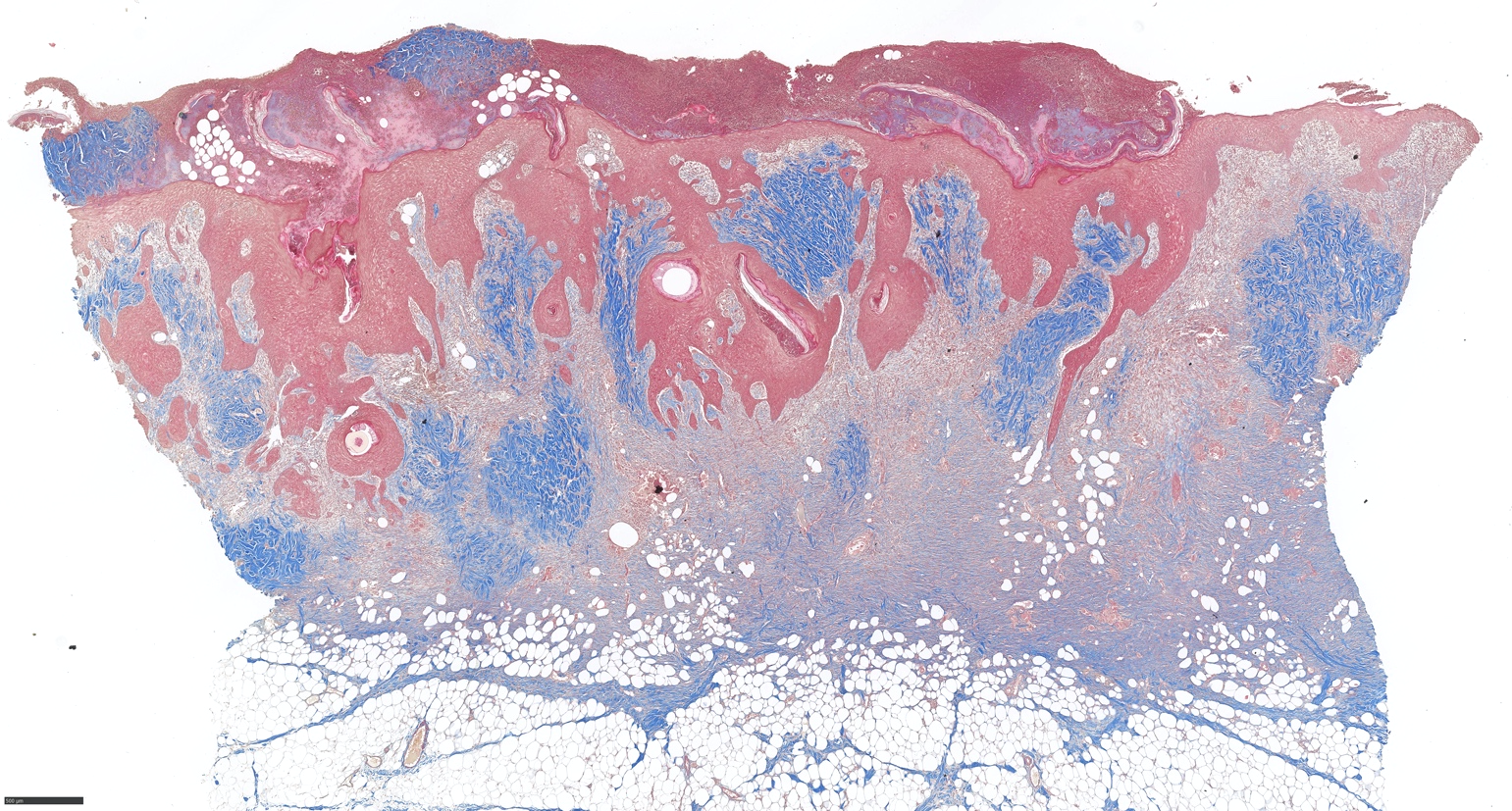


500 μm

***Supplementary Figure 5:* Whole-biopsy image of control wound at week 2:**

**
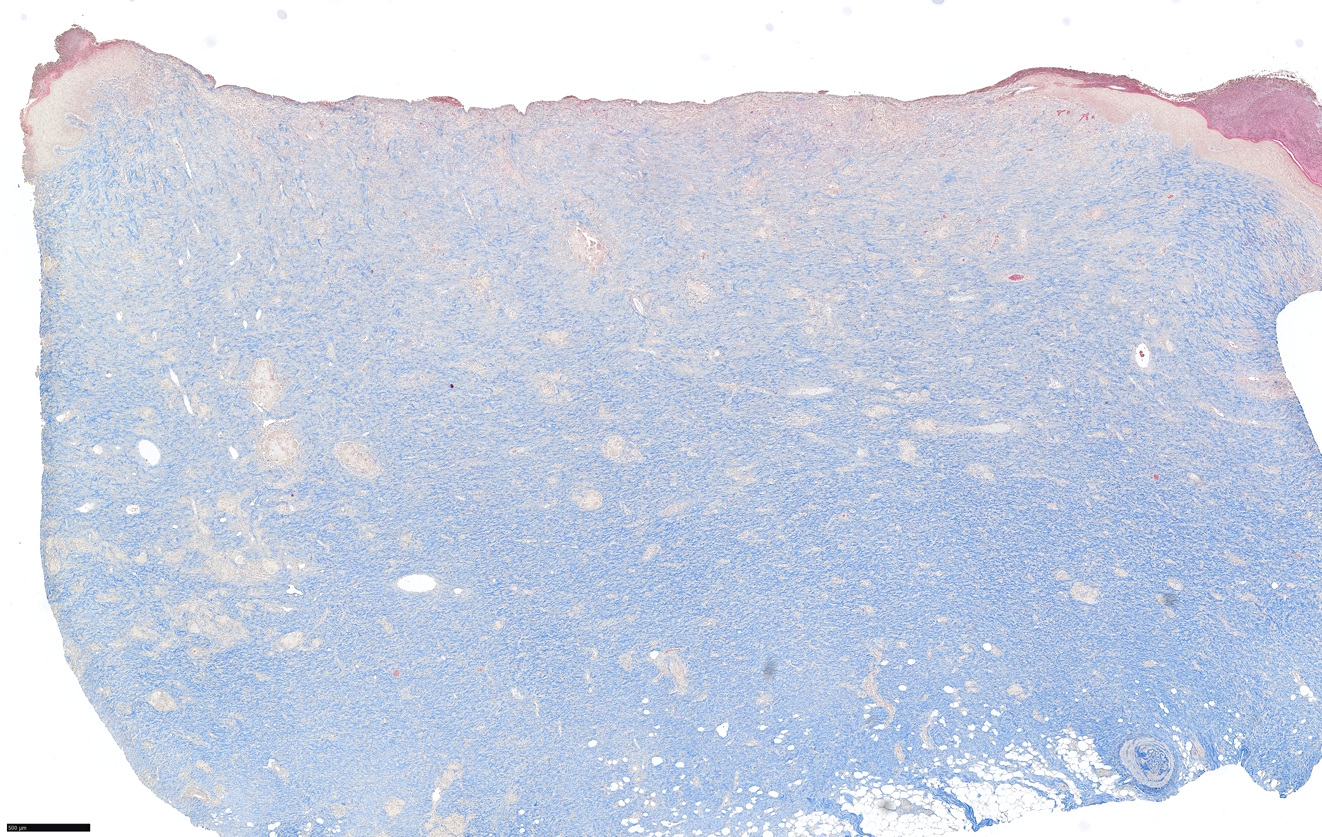
**

500 μm

***Supplementary Figure 6:* Whole-biopsy image of MSTC-treated wound at week 2:**


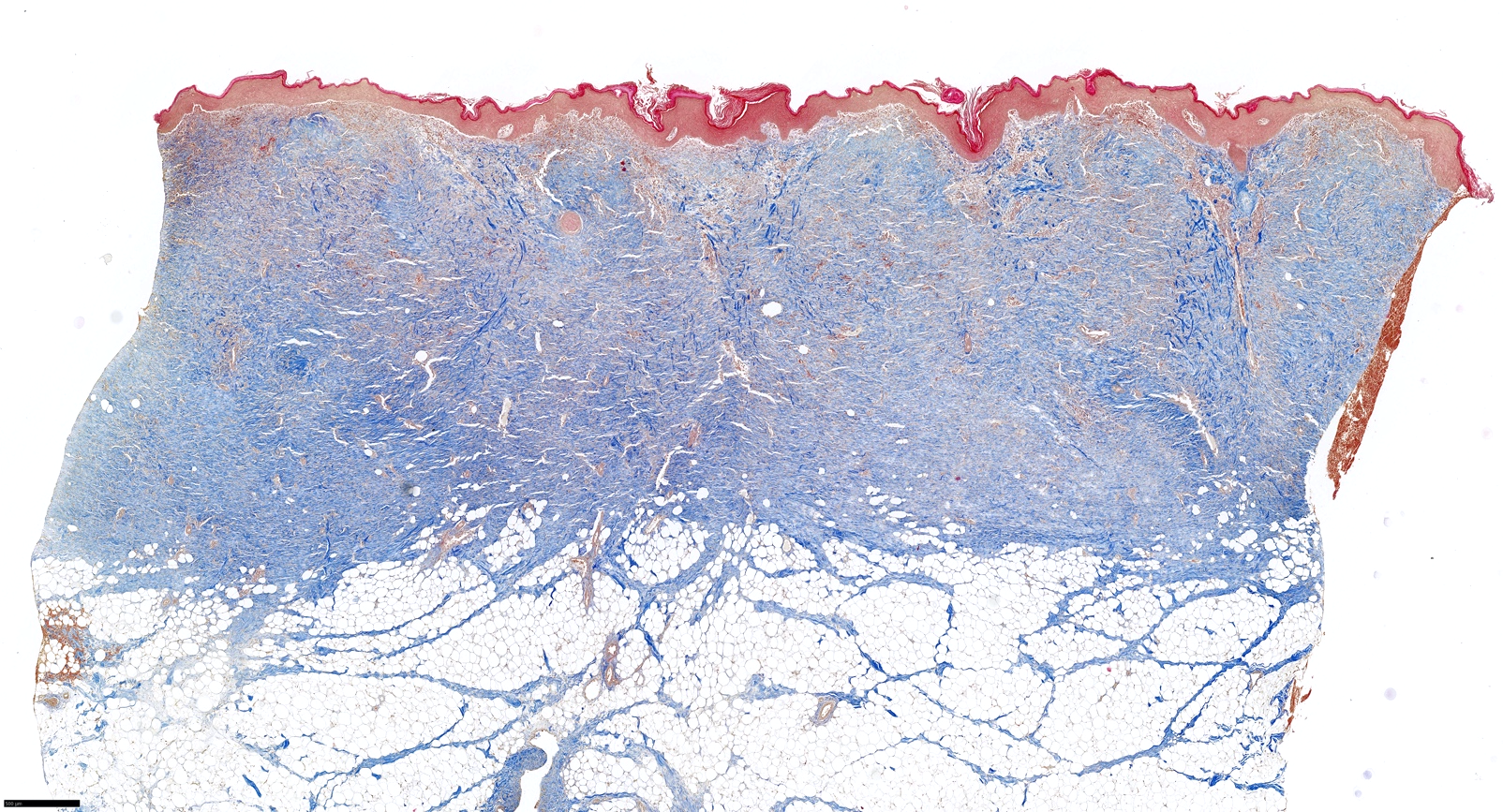


500 μm
